# Palatable Meal Anticipation in Mice

**DOI:** 10.1371/journal.pone.0012903

**Published:** 2010-09-30

**Authors:** Cynthia T. Hsu, Danica F. Patton, Ralph E. Mistlberger, Andrew D. Steele

**Affiliations:** 1 Broad Fellows Program in Brain Circuitry, Division of Biology, California Institute of Technology, Pasadena, California, United States of America; 2 Department of Psychology, Simon Fraser University, Burnaby, British Columbia, Canada; Vanderbilt University, United States of America

## Abstract

The ability to sense time and anticipate events is a critical skill in nature. Most efforts to understand the neural and molecular mechanisms of anticipatory behavior in rodents rely on daily restricted food access, which induces a robust increase of locomotor activity in anticipation of daily meal time. Interestingly, rats also show increased activity in anticipation of a daily palatable meal even when they have an ample food supply, suggesting a role for brain reward systems in anticipatory behavior, and providing an alternate model by which to study the neurobiology of anticipation in species, such as mice, that are less well adapted to “stuff and starve” feeding schedules. To extend this model to mice, and exploit molecular genetic resources available for that species, we tested the ability of wild-type mice to anticipate a daily palatable meal. We observed that mice with free access to regular chow and limited access to highly palatable snacks of chocolate or “Fruit Crunchies” avidly consumed the snack but did not show anticipatory locomotor activity as measured by running wheels or video-based behavioral analysis. However, male mice receiving a snack of high fat chow did show increased food bin entry prior to access time and a modest increase in activity in the two hours preceding the scheduled meal. Interestingly, female mice did not show anticipation of a daily high fat meal but did show increased activity at scheduled mealtime when that meal was withdrawn. These results indicate that anticipation of a scheduled food reward in mice is behavior, diet, and gender specific.

## Introduction

A near universal function of circadian clocks is to coordinate behavior and physiology with local environmental time as defined by the daily rising and setting of the sun [1]. In mammals, this function is critically dependent on a master, light-entrainable circadian pacemaker located in the hypothalamic suprachiasmatic nucleus (SCN) [2]. Circadian clocks in at least some species have been further exploited to enable animals to anticipate predictable daily opportunities to acquire food [3]–[7]. In rodents, the ability to behaviorally anticipate a restricted daily mealtime is mediated by a food-entrainable circadian mechanism that can be readily dissociated from SCN-driven activity rhythms, and that does not require an intact or functional SCN [8], [9].

The neural sites and molecular mechanisms of SCN-independent, food entrainable circadian oscillators have not been conclusively established. Involvement of the mesolimbic dopamine reward system in circadian anticipatory behavior is suggested by several observations, including 1. expression of the immediate early gene *c-fos* (signifying neural activation) and the clock gene *per1* (signifying circadian clock cycling) in the nucleus accumbens of food anticipating rats [10]–[12] and mice [13], 2. release of dopamine in the nucleus accumbens (Acb) in advance of a scheduled mealtime (C. Blaha and R. Mistlberger, unpublished observations), and 3. attenuation of food anticipatory activity rhythms in rats sustaining lesions of the Acb core [14], although not the Acb shell, or core and shell combined [15]. Highly palatable foods also elicit dopamine release in the Acb [16]–[18], and if provided as a snack at a fixed time of day can induce food anticipatory activity rhythms in rats with *ad libitum* access to regular chow [10], [19], [20]. This provides an alternate model for examining the role of reward systems in anticipatory behavior, and may be more appropriate for species, such as mice, that are physiologically less tolerant of circadian feeding schedules in which mealtimes are highly compressed, or calories severely reduced.

Mice anticipate restricted food access [21]–[23], and genetic models are available to test hypotheses concerning a role for the mesolimbic dopamine reward system. In addition, testing of mice with diminished food anticipatory activity [24] in another anticipatory model would be of great value in determining the circuit level inputs into the food-entrainable oscillator. However, it is not known if daily palatable meals, without caloric restriction, can entrain anticipatory rhythms in mice. Here we present results from two independent laboratories, using running wheels, motion sensors, video based analyses, and several palatable feeding schedules. Our results show that mice, unlike rats, surprisingly fail to exhibit robust and/or consistent anticipatory locomotor activity rhythms to a highly palatable midday meal, although they do exhibit modest anticipatory activity and searching directed at the food bin when a fat-enriched palatable food is provided.

## Materials and Methods

### Experiment 1. Wheel running activity measurement

Palatable meal experiments employing running wheels to assess anticipatory behavior were conducted at Simon Fraser University. Male C57BL/6J mice (N = 24, 19–21 g, ∼6 weeks old; Charles River, Quebec) were housed individually in opaque polypropylene cages (47×26×20 cm), in ventilated isolation cabinets (one mouse per compartment) with controlled lighting (LD 14∶10, ∼30 lux) and temperature (∼22+/− 1°C). A 14 h photoperiod was chosen based on a recent report that food anticipatory activity is more robust in mice maintained on long days [23]. Cages were equipped with stainless steel running wheels (17 cm diameter) and metal tops with a food hopper for LabDiet Laboratory Rodent Diet 5001 (28.5% protein, 13.5% fat, 58.0% carbohydrates) chow pellets and a water bottle. Corncob bedding was changed weekly. Wheel revolutions were detected by microswitches monitored continuously using the Clocklab (Actimetrics, IL) data acquisition system. For one week, a subset of cages (N = 7) were also equipped with infra-red motion sensors yielding a measure of total activity that included wheel running.

The mice were acclimated to the recording room and LD cycle for 3 weeks. Body weights and food intake were measured daily at lights-off during the last 2 weeks. The mice were then assigned in groups of 8 to one of three feeding schedules. Group 1 mice were food deprived for 20 h, beginning at Zeitgeber Time (ZT) 8 (i.e., 4 h before lights-off, defined as ZT12 by convention), and were then provided 3–4 g of regular chow pellets at ZT4 each day, adjusted to provide 75% of average daily ad libitum food intake for each mouse. The mice were weighed during the first hour of food access. After 14 days, regular chow was made available ad libitum, and ∼4 g of a highly palatable, nutritionally balanced food (Fruit Crunchies, 190 mg pellets, Bio-SERV; product # F05798, containing 6.2% fat, 20.2% protein, 52% carbohydrates by weight) was provided from ZT 4–6 daily for 28 days. Frunch Crunchies were provided in a tray placed inside the cage. Any uneaten Fruit Crunchies were removed. After day 28, the mice received ad libitum access to normal chow for one full day, and were then food deprived from ZT20 to ZT4, at which time they received regular chow, adjusted to 75% of ad libitum intake, each day for a final 17 days. Thus, Group 1 mice went from restricted feeding, to restricted palatable meal access with regular chow ad libitum, then back to restricted feeding.

Group 2 mice were provided with free access to regular chow and with restricted access to a supplementary palatable meal at ZT 4:00–6:00 each day for 67 days. The palatable meal consisted first of Fruit Crunchies for 39 days (3–4 g, 1 h/day for 11 days, then 2 h/day for 28 days) and then chocolate for 28 days (2.7 g, 2 h/day, Hershey's Milk Chocolate Chips, containing 33% fat, 6% protein, 53% carbohydrates, by weight).

Group 3 mice were provided with free access to regular chow and restricted access to a supplementary palatable meal from ZT4-6, consisting first of chocolate flavored Slimfast (Unilever, USA; containing ∼7% fat, 17% protein, 66% carbohydrates by dry weight, mixed in a ratio of 2.5 g powder/ml water) for 24 days and then Hershey's chocolate chips for 45 days. During the first 21 days of restricted chocolate meal access, the mice had free access to an unlimited amount of chocolate during the two hours of access while during the last 24 days, chocolate was limited to 1.4 g (estimated to be 35% of total daily calories), and regular chow was limited to 2–3 g, replenished daily at dark onset, to limit total daily caloric intake to ∼75% of ad libitum intake.

### Experiment 2. Video recording activity measurement

Palatable meal experiments employing video based assessment of anticipatory behavior were conducted at the California Institute of Technology. Male C57BL/6J mice (10–12 weeks of age, Jackson Labs West) were singly housed for 48–72 h prior to initiating the experiment. All mice were allowed ad libitum access to LabDiet Laboratory Rodent Diet 5001 and water and were entrained to a 13∶11 LD cycle at a temperature between 21–24°C. Light intensity during “lights on” was approximately 30 lumens/square foot in the husbandry room and 40 lumens/square foot in the video recording room. Mice were housed in clear polypropylene cages juxtaposed to each other such that they could see other mice both during normal housing as well as during video recordings. Mice were video recorded on days 0, 7, 14, 21, and 28 for 23.5 to 24 h (starting at ZT10, i.e., 2 h before lights-off). At the start of the day 0 recording, mice were divided into three groups: one group received a daily treat of a single Ghiradelli's milk chocolate chip (0.9 g±0.05 g, containing 6.7% protein, 26.2% fat, and 67.1% carbohydrates by weight), one group received a daily treat of 0.9 g (±0.05 g) of high fat diet (Bio-SERV; containing 35.5% fat, 20.0% protein, and 36.3% carbohydrates by weight), and the third group received only an additional 5001 chow pellet daily as a disturbance control. The meal was delivered in the food bin. This procedure was repeated for 28 days, with the mice receiving a chocolate chip, a small high fat meal, or an additional pellet at ZT10 each day. Regular 5001 chow was available ad libitum for all groups, including the palatable meal withdrawal experiment with the C57BL/6J females in experiment 2. Palatable meals, either chocolate or high fat, were always consumed entirely and remnants did not need to be removed from the cage or food bin.

Preference tests were conducted in a novel group of seven female and two male mice single-housed for 48 hrs prior to the test. Mice were given ad libitum access to food and water and given ad libitum access to Ghiradelli's chocolate and high fat diet between ZT 7:30 and 9:30 for three successive days.

To test the effect of gender, female C57BL/6J mice (8 weeks of age, Jackson Labs West) were singly housed for 48 – 72 h prior to initiating the experiment. All mice were allowed ad libitum access to LabDiet Laboratory Rodent Diet 5001 and water and were entrained to a 13∶11 LD cycle at a temperature between 21–24°C. Mice were video recorded on days 0, 7, 14, and 21 for 21.75 to 22 h (starting at ZT9, i.e., 3 h before lights-off). At ZT 7, the recordings were paused for about fifteen minutes for feeding, after which the mice were recorded 1.75 to 2 hours. One group received a daily meal of 0.8 g (±0.05 g) of high fat diet (approximately 25% of controls' day 0 food intake) and the control mice received an additional 5001 chow pellet daily as a disturbance control. Following the feeding at ZT 7 on day 28, both ad libitum and high fat diet mice were recorded undisturbed for 48 hours. No additional food (either high fat or standard chow) was added to their cages at this time, although an *ad-libitum* amount of food and water was available to the mice through the entire duration of the recording.

All experiments were approved by the respective institutional animal care committees of Simon Fraser University and California Institute of Technology (protocol number 1567 and protocol number 935p) and efforts were made to reduce the number of animals used in these studies.

### Data analysis

Wheel running and motion sensor activity data acquired using Clocklab were collapsed into 10 min bins and plotted in standard actogram and average wave formats using Circadia (Dr. T. Houpt, Florida State University) and Prism 5.0 (GraphPad Software, Inc., La Jolla, CA).

Video based activity data were analysed using HomeCageScan 3.0. Behavioral definitions were as described previously [25]. High intensity activity was defined as walking, jumping, rearing, and hanging behaviors. Food bin activity was defined as how often the nose of the mouse entered the manually labeled food bin. Activity data were accumulated into 1 h time bins, and evaluated statistically using the Tukey-Kramer Multiple Comparisons Test with Repeated Measures or one-way ANOVA post-tests as appropriate, or the Dunn's test with Kruskal-Wallis post-test (GraphPad Prism 4). Medians are reported + interquartile ranges. Total high intensity activity was also output from HomeCageScan 3.0 and defined as the frames in which jumping, rearing, and hanging was observed during the entire recording period divided by the total number of frames.

## Results

### Experiment 1: Running wheel activity in mice receiving Fruit Crunchies, chocolate or Slimfast

**Figure 1:**
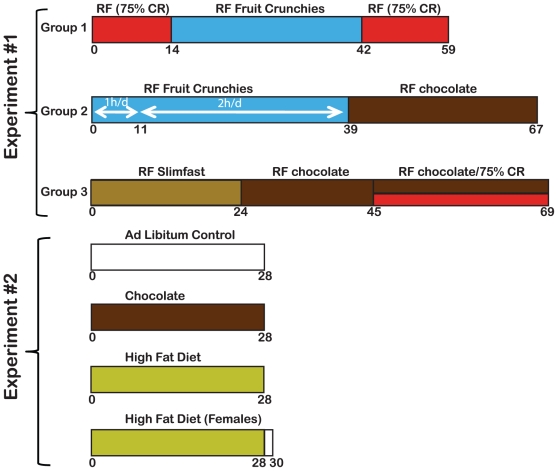
Diagrammatic representation of experiments. “Experiment #1” comprises conditions used at Simon Fraser University measuring activity mainly with running wheels and “Experiment #2” comprises conditions used at Caltech using computer vision to assess activity. Days of treatments are indicated as numbers below the colored bars, red represented calorie restriction, blue for fruit crunchies, brown for chocolate, white for no treatment, and yellow for high fat diet. For experiment #1, mice were fed the palatable snack from ZT4-6. For experiment #2, male mice were provided with the palatable snack at ZT10 and the snack was not removed; for female mice the snack was presented at ZT7. RF, restricted feeding; CR, calorie restriction.

**Figure 2:**
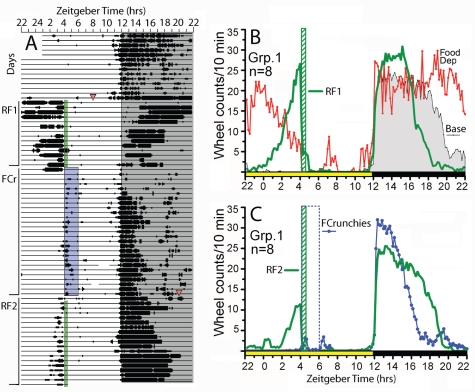
Wheel running activity of mice in Experiment 1, Group 1. (A) Actogram of wheel running from one representative mouse. Time of day in 10 min bins is plotted left to right, and consecutive days are aligned vertically. Time bins during which no wheel revolutions occurred are represented by a single point. Bins during which revolutions were registered are represented by vertical bars (3 points = 1–10 counts, 5 pts =  11–20 counts, 7 pts = >20 counts/10 min). Lights-off is indicated by grey shading (ZT12-22). Vertical bars to the left indicate experimental conditions, as follows: RF1, restricted feeding schedule 1, regular chow provided at ZT4 each day (denoted by green shaded box), adjusted to 75% of ad libitum intake; FCr, ad libitum access to regular chow, with time limited access to Fruit Crunchies from ZT4-6 (blue shaded box); RF2, a repeat of the first restricted feeding schedule (green shaded box). (B) Group mean waveforms of wheel running during ad libitum food access prior to restricted feeding (Base, grey fill, 7-day average), during 20 h of food deprivation starting at ZT8, prior to restricted feeding (Food Dep, red line), and during first restricted feeding schedule (RF1, green line, last 7 days). (C) Group mean waveforms of wheel running during restricted access to Fruit Crunchies (FCr, blue line, last 7 days) and during the second restricted feeding schedule (RF2, green line). The timing of lights on and off is indicated by the yellow and black bars, respectively, along the abscissa.

**Figure 3:**
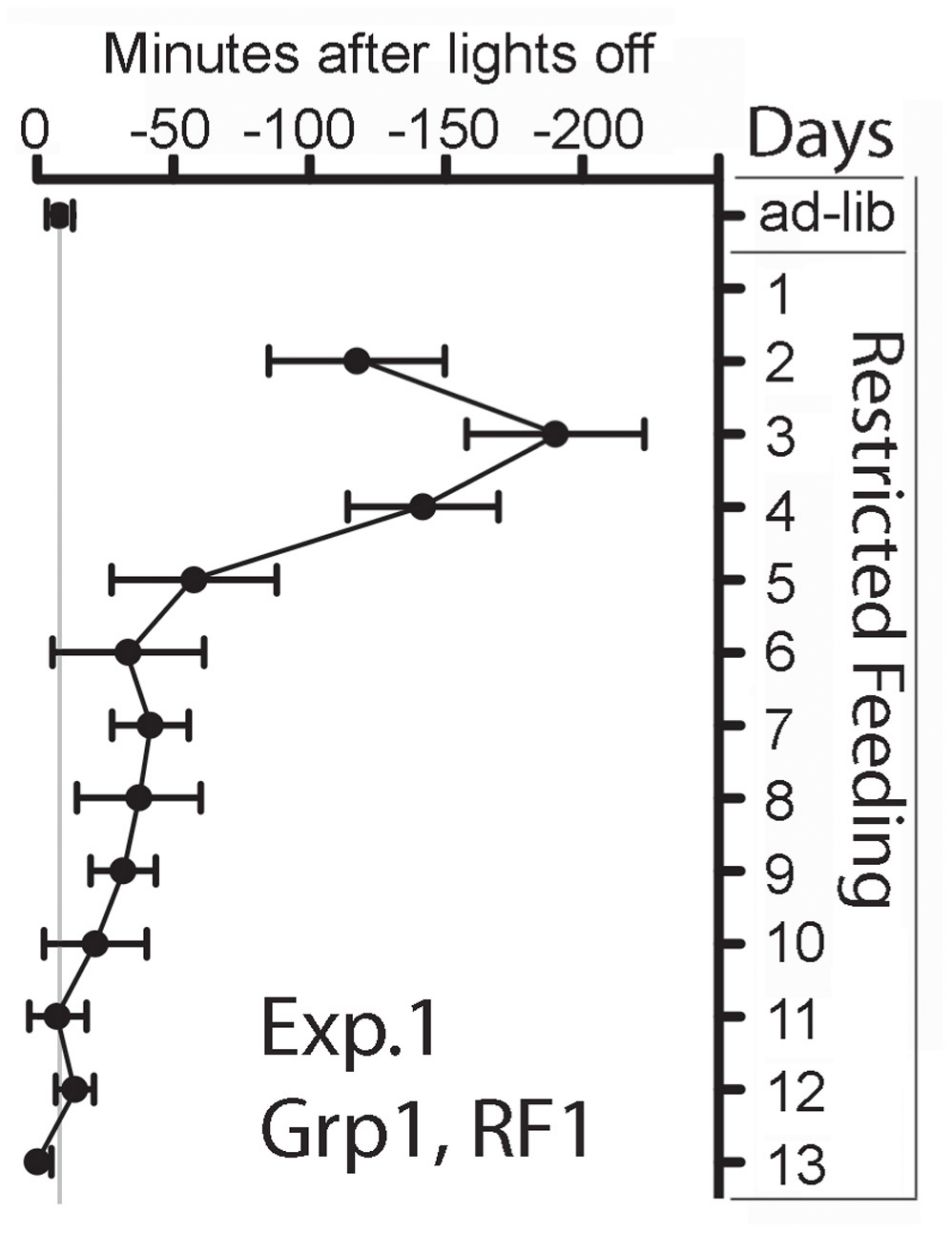
Nocturnal wheel running onset for Experiment 1, Group 1. Mean (± SEM) time of nocturnal wheel running onset during the last week of *ad libitum* food access (7 day mean), and for each day of the first restricted feeding schedule. Onset time is expressed in minutes relative to lights-off. All mice were hypoactivity following the first meal, resulting in no nocturnal wheel running onset on day 1. Onsets on the next 4 nights were delayed relative to nocturnal activity onset during *ad libitum* food access.

A diagramatic representation of all experimental conditions is provided in **Figure 1**. Prior to scheduled daytime feeding, wheel running activity was concentrated during the first 6–8 h of the 10 h dark period, declined during the last 2–4 h of dark, and was virtually absent during the light period, in all three groups of mice. When Group 1 mice were food deprived for 20 h, nocturnal wheel running remained elevated throughout the night and declined gradually from light onset until the first scheduled meal at ZT4 (**Fig. 2a–b**). Body weights after the 20 h fast were reduced by 20%±4% from their initial starting weights of 22.6±1.4 g (mean + SD), but gradually recovered to baseline levels by day 14 of restricted feeding. All Group 1 mice exhibited robust food anticipatory running, beginning 2–4 h prior to mealtime (**Fig. 2a–b**). On the night following the first scheduled daytime meal, nocturnal activity was markedly reduced in all 8 mice (e.g., **Fig. 2b**). Nocturnal running reappeared on the second night, but the onset was delayed by 125±91 min relative to the onset of nocturnal running during ad libitum food access (**Fig. 3**). On subsequent nights, nocturnal activity onset gradually advanced toward ZT12, reaching dark onset within 5–10 days (e.g., **Figs. 2a** **,** **3**). By the last week of restricted feeding, nocturnal activity was compressed to within the first 4 h of the dark period.

On day 14 of restricted feeding, Group 1 mice received Fruit Crunchies at ZT4 for 2 h, and chow was made available ad libitum. Food anticipatory running disappeared immediately in all cases and remained absent for the entire 28 days of scheduled palatable food access (**Fig. 2a, c**). Two hour intake of Fruit Crunchies averaged 3.4±.2 g during the first day of access, but declined over the first week in all mice, stabilizing at about 1.5±.2 g over the last 2 weeks. Nocturnal activity was strongly decreased for several days in all mice, but gradually recovered (**e.g.,** **Fig. 2a**). Body weights increased by 26±7% over the 28 days of palatable food access. When food was again restricted to 75% of ad libitum intake, replenished daily at ZT4 without Fruit Crunchies, food anticipatory activity re-emerged, although more gradually than during the first restriction schedule and with a lower peak level (e.g., **Fig. 2c**). The mice lost 8±1% body weight on the first day of food restriction, and remained at about this level over the next 17 days.

**Figure 4:**
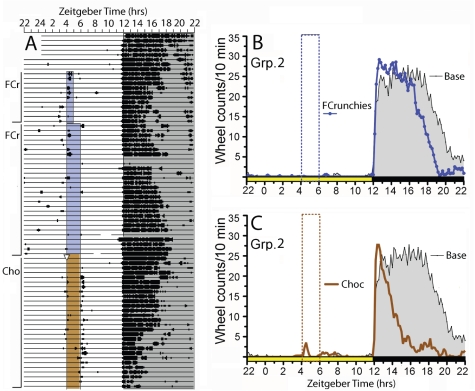
Wheel running activity of mice in Experiment 1, Group 2. (A) Actogram of wheel running from one representative mouse. Vertical bars to the left indicate experimental conditions, as follows: FCr, *ad libitum* access to regular chow, with time limited access to Fruit Crunchies from ZT4-5 (blue shaded box); FCr, *ad libitum* access to regular chow, with time limited access to Fruit Crunchies from ZT4-6 (blue shaded box); Cho, *ad libitum* access to regular chow with time limited access to Hershey's Milk Chocolate Chips (brown shaded box). (B) Group mean waveforms of wheel running during *ad libitum* food access prior to restricted feeding (Base, grey fill, 7-day average), and during the last 7 days of time limited access to Fruit Crunchies (FCrunchies, blue line). (C). Group mean waveforms of wheel running during last 7 days of restricted access to chocolate (Choc, brown line). Vertical boxes indicated feeding or snack time.

Group 2 mice were provided restricted access to Fruit Crunchies for 39 days and then restricted access to chocolate chips for 27 days from ZT4-6 (**Fig. 1**). Similar to Group 1 mice, none of the mice in Group 2 exhibited anticipatory wheel running to Fruit Crunchies or chocolate chips (e.g., **Fig. 4a-c**). Intake of Fruit Crunchies increased gradually over the first 10 days before stabilizing at 1.4±.1 g/day (1.3–1.6 g/day across mice). Chocolate intake (days 40–66) averaged 1.2±.1 g/day (1.1 – 1.4 g/day across mice). The mice gained 14±3% body weight during access to Fruit Crunchies, and another 2±3% during access to chocolate.

**Figure 5:**
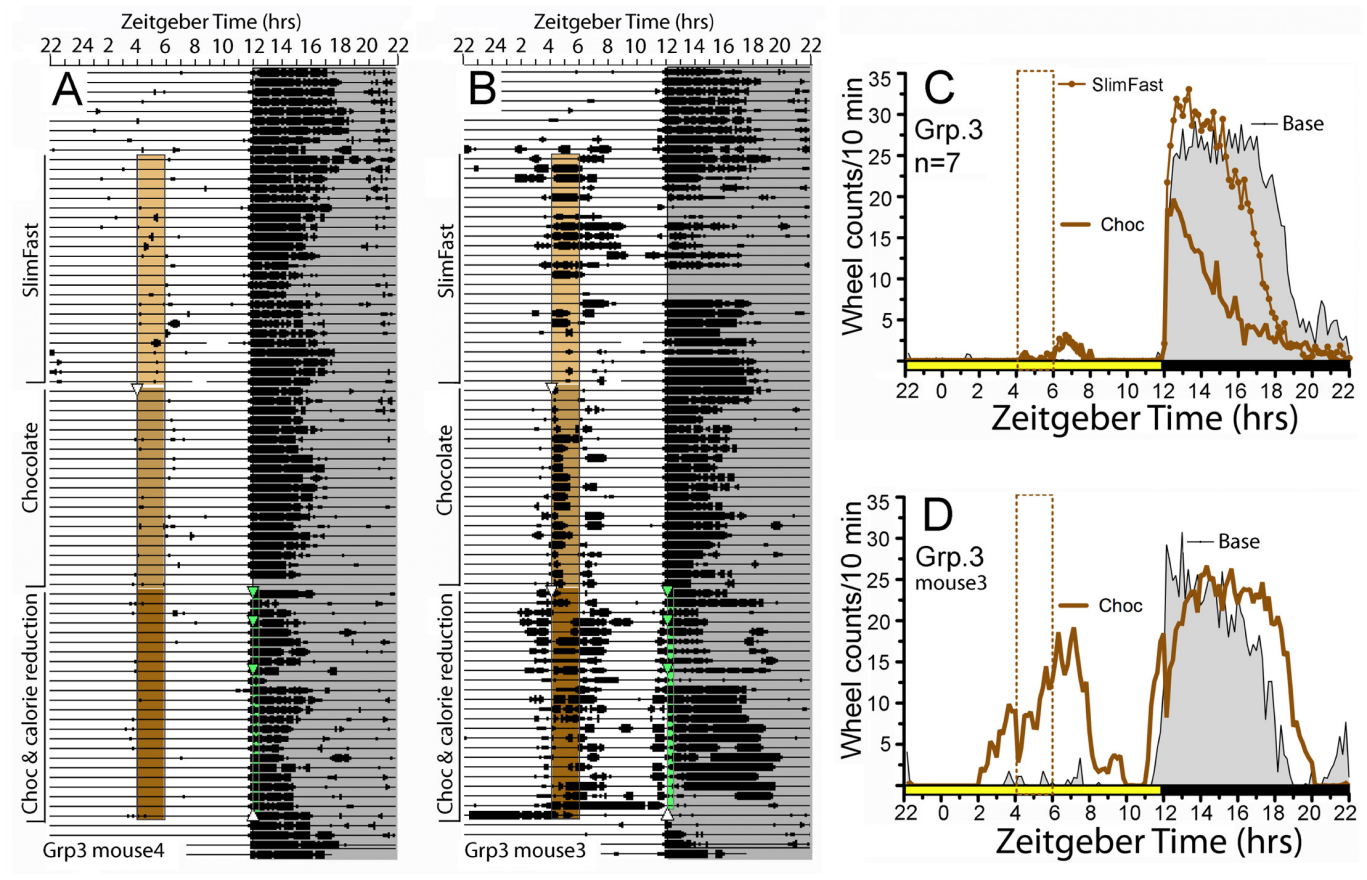
Wheel running activity of mice in Experiment 1, Group 3. (A) Actogram of wheel running from one representative mouse. Vertical bars to the left indicate experimental conditions, as follows: SlimFast, *ad libitum* access to regular chow, with time limited access to a chocolate flavored SlimFast paste from ZT4-6 (light brown shaded box); Chocolate, *ad libitum* access to regular chow, with time limited access to Hershey's Chocolate Chips from ZT4-6 (medium brown shaded box); Chocolate Chips from ZT4-6 (dark brown shaded box) with regular chow replenished daily at ZT12 (green shaded box), in gradually decreasing amounts (green arrowheads denoted reductions, down to 75% of *ad libitum* intake). (B) Actogram of the one mouse in this group that exhibited anticipatory running to time limited chocolate access. (C) Group mean waveforms of wheel running during *ad libitum* food access prior to restricted feeding (Base, grey fill, 7-day average), during the last 7 days of time limited access to SlimFast (brown line with circles) and during the last 7 days of access to Chocolate (Choc, heavy brown line). (D) Average waveforms of wheel running from the one mouse that anticipated chocolate access, when this was combined with caloric restriction.

Group 3 mice were provided access to Chocolate Slimfast for 24 days and then to chocolate chips for another 21 days for 2 h/day at ZT4, with chow freely available (**Fig. 1**). The mice consumed on average 1.1±.2 g of Slimfast, and 1.4±.1 g of chocolate but did not exhibit wheel running prior to palatable meal time (Fig. 5). Body weight increased by 10±4% over this time. When regular chow was then gradually reduced over a 9 day period to 2 g/day, replenished each day at dark onset, one mouse within 2 days began to run prior to chocolate at ZT4 and again prior to chow replenishment at ZT12 (**Fig. 5b,d**). This mouse ate 1.4 g of chocolate/day (>35% of total daily caloric intake) and lost 18% body weight over the first 23 days of reduced caloric intake. On the last day, this mouse did not receive chocolate, and exhibited continuous running from ZT4 to dark onset, and then again the next day from light onset to ZT4 (i.e., all of its running was diurnal). The other 7 mice in this group did not show anticipatory running to either daytime chocolate or nocturnal chow replenishment (e.g., Fig. 5a,b). These mice ate 1.3±.1 g of chocolate/day and exhibited more moderate weight changes, in the 8% to +8% range.

**Figure 6:**
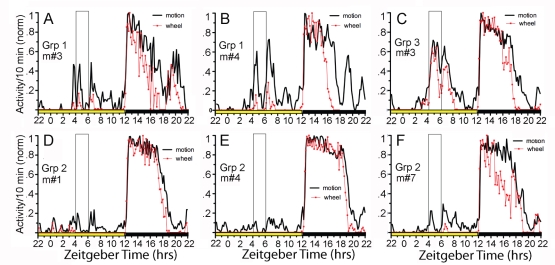
Activity measurements taken with infra-red motion sensors. Waveforms of locomotor activity (normalized) measured concurrently by infra-red motion sensors (heavy black curves) and running wheels (thin red curves), averaged over one week, from individual mice in (A–B) Group 1, (D–F) Group 2, and (C) Group 3. The daily palatable mealtime is denoted by the rectangles (hours 6–8 of lights-on).

To assess whether the mice might be anticipating palatable food access despite the absence of anticipatory wheel running, motion sensors were installed over 7 cages (one from Group 1 and three each from Groups 2 and 3) for one week, after intake of palatable food had stabilized. In each case, some pre-palatable meal activity was evident, but it was sporadic and very low relative to nocturnal activity in the same mice, and to food anticipatory running in Group 1 mice (**Fig. 6**).

### Experiment 2: Automated video analysis of home cage behavior in mice receiving palatable meals of chocolate and high fat

**Figure 7:**
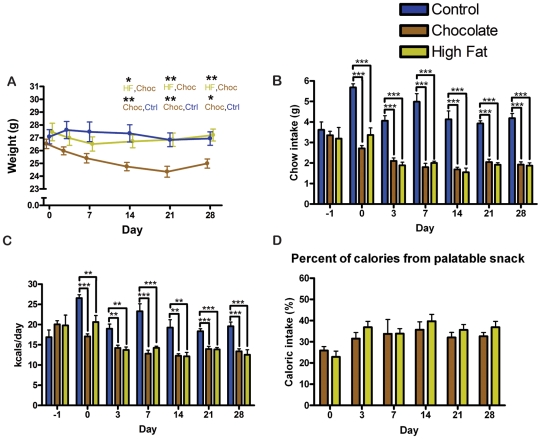
Body weight, food intake, and caloric intake of male mice in experiment 2. (A) Male mice fed chocolate show a significant decrease in weight relative to those fed ad libitum or high fat meals. Male mice fed high fat meals do not show a change in weight relative to ad libitum controls. (B) Male mice fed chocolate and those fed high fat show a significant decrease in normal chow intake in comparison to mice with ad libitum access. (C) Male mice fed chocolate and those fed high fat show a significant decrease in total caloric intake (the sum of the normal chow plus the palatable meal) in comparison to mice with *ad libitum* access. Caloric intake values were estimated from nutritional facts provided by the respective companies. (D) The percent of total caloric intake per day provided by the palatable meal. Bars represent means±SEMS and the statistical test was Tukey-Kramer Multiple Comparisons Test with One-Way ANOVA post-test, * denotes p<0.05, ** denotes p<0.01, and *** denotes p<0.001.

Unlike Group 2 and 3 mice in Experiment 1, mice in Experiment 2 lost weight when fed chocolate once daily, and weighed significantly less on days 7, 14, 21, and 28, relative both to their starting weight (p<0.001) and to the control group (p<0.05) (Fig. 7A). The body weight of mice fed a small high fat meal daily did not differ from control mice, and was significantly lower than starting weight (day 0) only on day 7 (p<0.05) (Fig. 7A). The control group had no significant changes in weight over time.

**Figure 8:**
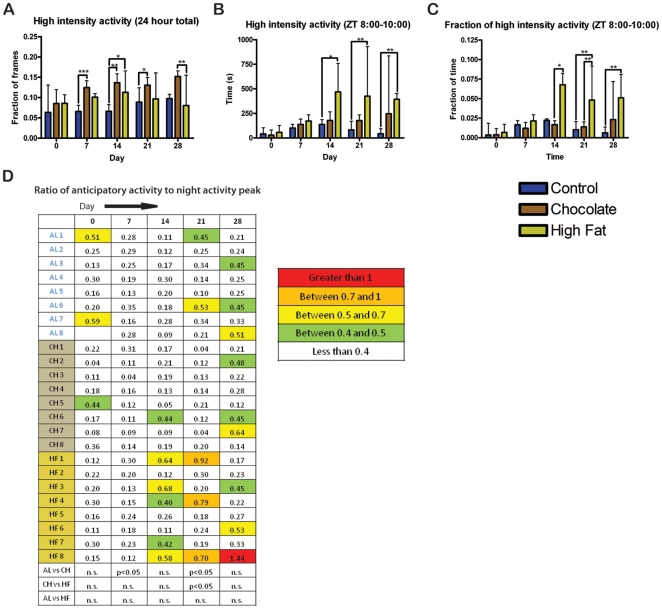
Home cage activity of male mice in Experiment 2. (A) The fraction of frames during the entire 23.5 to 24 h recording period during which the mice were walking, hanging, jumping, and rearing. Male mice receiving daily chocolate are significantly more active than *ad libitum* controls on days 7, 14, and 21. Mice receiving daily high fat are significantly more active than ad libitum controls on day 14. (B) The total seconds of walking, hanging, jumping, and rearing during the 2 h before the palatable meal is received. (C) For each individual mouse the sum of the total seconds of walking, hanging, jumping, and rearing during the 2 h before the palatable meal is divided by the total seconds spent walking, hanging, jumping, and rearing during the 23.5 to 24 h recording period. The mice fed high fat show a significantly greater difference in the fraction of high activity during this 5 h period than the mice fed chocolate. (D) The maximum number of seconds per hour of walking, hanging, jumping, and rearing in the 5 h preceding feeding is divided by the maximum number of seconds per hour of walking, hanging, jumping, and rearing at night. Ratios greater than 1 are shown in red, between 0.7 and 1 in orange, between 0.5 and 0.7 in yellow, and between 0.4 and 0.5 in green. Bars show medians and interquartile ranges. Statistical test was Kruskal-Wallis test with Dunn's post-test, * denotes p<0.05, ** denotes p<0.01, and *** denotes p<0.001.

Both chocolate and high fat meals were rapidly consumed by the mice, usually within a half hour. Chow intake was significantly and consistently decreased in both groups (high fat and chocolate) receiving a daily palatable meal (p<0.001) (Fig. 7B). Surprisingly, the total caloric intake (calories from chow plus palatable meal) was also significantly decreased in both daily chocolate and high-fat fed groups relative to controls (p<0.01) (Fig. 7C). Calories from the palatable meal were roughly the same for mice receiving high fat and mice receiving chocolate, between 18.6% and 44.7% of their daily caloric intake on day 0 of the study and ranging from 25.2% to 46.1% of their daily caloric intake on subsequent days (Fig. 7D). Mice receiving daily chocolate exhibited a statistically significant increase in overall high intensity activity over the entire 24 h recording period in comparison to controls at days 7, 14, 21, and 28 (p<0.05). Mice receiving a daily high fat treat were also significantly more active on days 14 and 28 (p<0.05) (Fig. 8A). High intensity activity summed over (2 h) prior to feeding shows that mice receiving high fat meals had significantly greater activity than controls at day 14, 21, and 28 of the experiment (Fig. 8B). This difference is also significant when the seconds of high intensity activity during the 2 h prior to feeding is normalized by dividing by the total number of seconds (Fig. 8C). To compare individual variations in anticipatory activity, the maximum number of seconds of high intensity activity per hour in the 5 h preceding feeding was divided by the maximum number of seconds of high intensity activity per hour during the dark cycle (Fig. 8D). Only one individual, a mouse receiving daily high fat, displayed high intensity activity comparable to the night time peak consistently at day 14, 21, and 28, although several mice also on the high fat diet displayed high intensity activity at one or two time points.

**Figure 9:**
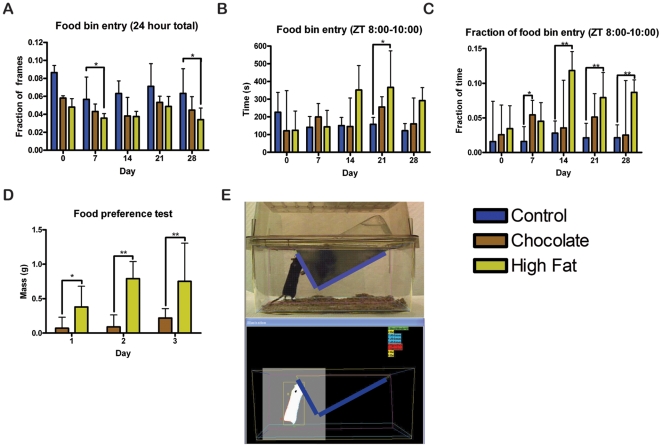
Food bin entry behavior of male mice in Experiment 2. (A) The fraction of frames during the entire 23.5 to 24 h recording period during which the mice had their nose in the food bin. (B) The total seconds with noses in the food bin during the 2 h before the palatable meal is received. (C) For each individual mouse the sum of the total seconds with their noses in the food bin during the 2 h before the palatable meal is received is divided by the total seconds spent with their noses in the food bin during the 23.5 to 24 h recording period. The mice fed high fat show a significantly greater difference in the fraction of high activity during this 2 h period than the mice fed chocolate. (D) Food preference test comparing chocolate and high fat food intake when both are available for 2 hours on three consecutive days. Normal chow was still available during the preference test. (E) Screenshot of a mouse eating in home cage scan with the food bin zone highlighted in blue. Statistical test was Kruskal-Wallis test with Dunn's post-test, where * denotes p<0.05, ** denotes p<0.01, and *** denotes p<0.001.

Because of the decreased total caloric intake, we also examined the number of seconds the mice spent with their nose in the food bin to examine eating and food seeking habits (see Fig. 7G for illustration). Mice that received a small high fat meal daily spent less total time with their snout in the food bin than did controls at all time points, but only significantly so at days 7 and 28 (p<0.05) (Fig. 9A). The number of seconds of food bin entry during the 2 h prior to receiving the palatable meal is significantly greater for high fat mice in comparison to the controls (p<0.05) (Fig. 9B). When the number of seconds of food bin entry during the two hours prior to palatable meal access is divided by the total seconds of food bin entry, high fat mice have a significantly greater fraction of food bin entry at day 14, 21, and 28. By comparison, mice fed chocolate have a significantly greater food bin entry fraction only on day 7 (Fig. 9C).

To determine if high fat was more palatable than chocolate, we performed a preference test. Mice were given simultaneous access to high fat diet and chocolate (in addition to normal chow) for two hours for three days in succession. Remarkably, on all three days of testing, high fat consumption was significantly greater than that of chocolate (Fig. 9D).

**Figure 10:**
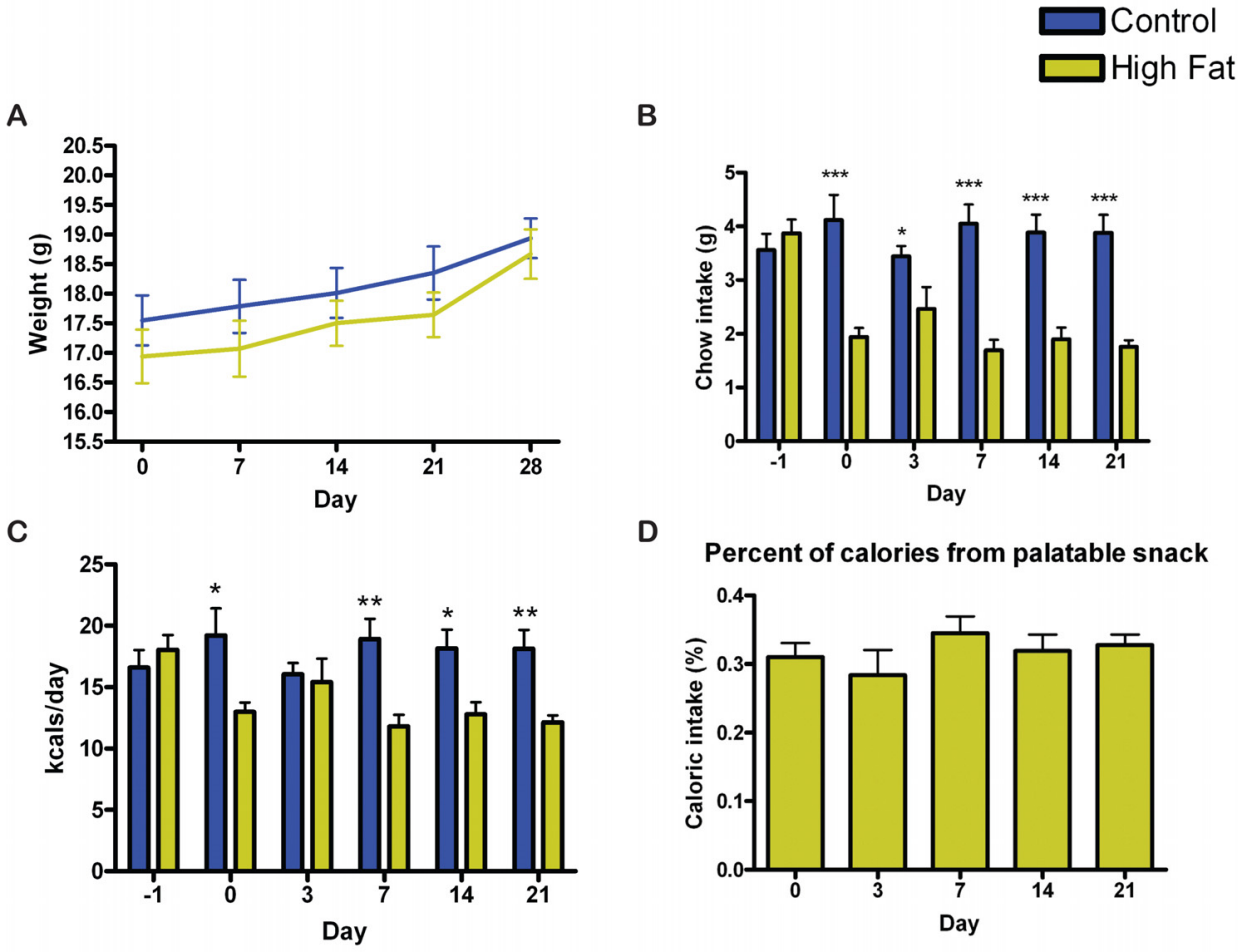
Body weight, food intake, and caloric intake of female mice in Experiment 2. (A) The mean (± SEM) body weight of female mice receiving 0.8 g of high fat daily (yellow) and their respective controls (blue). (B) Female mice fed high fat show a significant decrease in normal chow intake in comparison to mice with ad libitum access. (C) Female mice fed high fat show a significant decrease in total caloric intake in comparison to mice with *ad libitum* access. Caloric intake values were estimated from nutritional facts provided by the manufacturers. (D) The percent of total caloric intake per day provided by the palatable meal. Unpaired t-test * denotes p<0.05, ** denotes p<0.01, and *** denotes p<0.001.

Finally, to test the effect of gender on anticipation of a daily high fat meal, we gave C57BL/6J female mice a 0.8 g meal of high fat diet at ZT 7:00 daily for 28 days, followed by a withdrawal of the palatable treat for 48 hours starting on day 28 (Fig. 1). As with the male mice, there were no significant differences between the body weights of mice receiving a daily high fat treat relative to the control, although both control and once daily high fat female mice showed an increase in weight over the duration of the experiment (Fig. 10A). Similar to male mice, the amount of chow intake decreased in female mice receiving daily high fat in comparison to control mice (p<0.05) (Fig. 10B) The estimated daily caloric intake of the female mice receiving a once daily high fat treat was also greatly reduced (p<0.001) on days 0, 7, 14, and 21, with no significant difference on day 3 (Fig. 10C). The palatable high fat meal accounted for approximately 30% of their total caloric intake (Fig. 10D).

**Figure 11:**
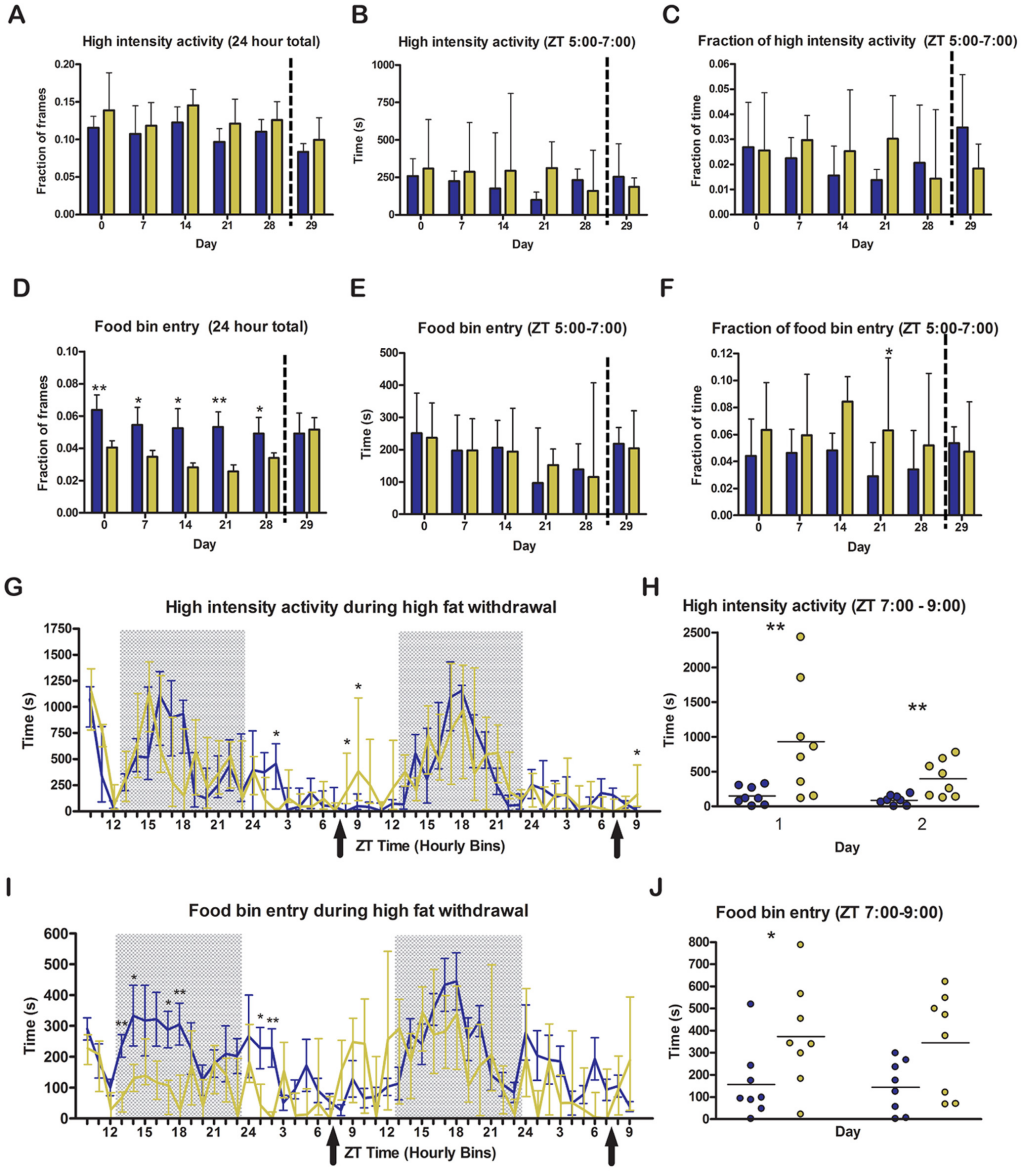
Home cage activity and food bin entry of female mice in Experiment 2 during palatable meal access and withdrawal. (A) The fraction of all recorded frames within a 24 hour period during which the female mice were walking, hanging, jumping, or rearing. There were no significant differences in the fraction of frames during which the female mice exhibited high intensity activity. (B) The total seconds of walking, hanging, jumping, and rearing during the 2 h before the palatable meal is received. There is no significant difference between the female mice receiving a once-daily palatable meal and controls. (C) For each individual mouse the sum of the total seconds of walking, hanging, jumping, and rearing during the 2 h before the palatable meal is delivered divided by the total seconds of walking, hanging, jumping, and rearing during both recording periods within a 24 hour window. There is no significant difference between the female mice receiving a once-daily palatable meal and those that are disturbed by a pellet of normal chow. (D) The fraction of all recorded frames within a 24 h period during which the female mice inserted their nose into the food bin. The mice spent significantly less time with their nose in the food bin on all recorded days (day 0, 7, 14, 21, and 28) with the exception of the second day of withdrawal of the palatable meal. (E) The total seconds of food bin entry during the 2 h before the palatable meal is received. There is no significant difference between the female mice receiving a once-daily palatable meal and those that are disturbed by a pellet of normal chow. (F) For each individual mouse the sum of the total seconds of food bin entry during the 2 h before the palatable meal is divided by the total seconds of food bin entry during both recording periods within a 24 h window. There is only a significant difference between the female mice receiving a once-daily palatable meal and those that are disturbed by a pellet of normal chow on day 21 of the special feeding regimen. (G) The total sum of seconds of walking, hanging, jumping, or rearing observed during each hour of the 48 hour high fat withdrawal. Shaded boxes represent lights off; arrows represent expected meal time. (H) Sum of high intensity activity during the two hours following expected palatable treat access on day 1 and 2 of withdrawal. High fat entrained mice show more high intensity activity than ad libitum controls on both days of the withdrawal. (I) Total sum of seconds of food bin entry observed during each hour of the 48 hour high fat withdrawal. Shaded boxes represent lights off; arrows represent expected meal time. (J) Sum of food bin entry during the two hours following expected palatable treat access on day 1 and 2 of withdrawal. High fat entrained mice show more food bin entry than ad libitum controls on both days of the withdrawal, although it is only significantly greater on the second day. Arrows indicate the bin in which palatable meal would have been delivered. Bars show medians and interquartile ranges; the statistical test used was Mann-Whitney where * denotes p<0.05, ** denotes p<0.01, and *** denotes p<0.001. Note that no palatable meals were administered on day 28 and 29 (equivalent to withdrawal days 1 and 2), but all mice retained ad libitum access to food and water.

Surprisingly, female C57BL/6J mice receiving a high fat meal daily showed no difference in their total high intensity activity (Fig. 11A), the total number of seconds of high intensity activity in the two hours preceding palatable treat access (Fig. 11B), and normalized high intensity activity (high intensity activity in the two hours preceding palatable treat access divided by the total high intensity activity) (Fig. 11C). In contrast to males, there was a significant decrease in total food bin entry over all days of the experiment, with the exception of day 29 (during which the mouse has undergone 48 hour palatable meal deprivation) (Fig. 11D). There was no difference in the total seconds of food bin entry during the two hours prior to palatable treat delivery (Fig. 11E). When the seconds of food bin entry during two hours prior to palatable treat delivery is divided by the total seconds of food bin entry, the value for high fat is significantly greater on day 21 only (11F).

Despite the lack of anticipatory activity, when palatable meals are withdrawn from the high fat access female mice for forty-eight hours while still permitting the mice ad libitum access to normal chow, many mice demonstrated an increase in both high intensity activity (Fig. 11G) and food bin entry (Fig. 11I) during and after the expected palatable meal time despite ad-libitum access to normal chow. When summed over the two hours following expected meal time, high intensity activity for mice that had previously received once daily high fat treat was significantly greater than that for mice fed ad-libitum on the first and second day of withdrawal, while food bin entry was significantly increased on the first day of withdrawal but not on the second day (Fig. 11H, 11J).

## Discussion

Rodents fed at the same time each day, for a limited duration (typically 2–4 h) or in a limited amount (e.g., 75% of ad libitum daily caloric intake), exhibit a robust increase of wheel running [3]–[5], general locomotor activity [12]–[14], and food-bin approaches [3], [7], [15], [26], [27] beginning 1–3 h before mealtime. This daily rhythm exhibits formal properties of a clock-controlled process, and has been conceptualized as the output of a food-entrainable circadian oscillator [3]–[5]. Rats with ad libitum access to regular chow also exhibit running or locomotor activity in anticipation of a highly palatable treat, such as a piece of chocolate [10], [12] or powdered chow mixed with chocolate syrup and corn oil [19]. Mice exhibit similar daily rhythms of food anticipatory running and general locomotor activity when food is temporally restricted [21], [23], and we expected that they would also anticipate a sweet and/or fatty snack, if the food was sufficiently palatable to stimulate eating during the lights-on period without caloric restriction. Surprisingly, although mice in both Experiments 1 and 2 ate the palatable snacks, most mice failed to consistently exhibited anticipatory locomotor activity. Furthermore, mice (Group 1) in Experiment 1 exhibited robust food anticipatory running when calorically restricted, but this anticipatory behavior was not sustained when chow was provided ad libitum and a palatable rodent diet (Fruit Crunchies) was provided at the usual mealtime. Only one mouse exhibited anticipatory running to a daily chocolate treat, and only when total daily calories were limited to 75% of ad libitum intake (Group 3). These results were corroborated using infra-red motion sensors that measure total activity in the cage.

Similar results were obtained using chocolate in a second experiment measuring activity with automated video-based behavioral analysis. With the exception of one mouse on a high fat diet, high intensity activity did not consistently rise to a comparable level to the night time activity peak as is commonly observed in restricted feeding experiments; however, they demonstrated significantly more high intensity activity during the 2 h prior to feeding than the ad libitum controls. Notably, mice receiving a high fat meal also exhibited fairly strong anticipatory food bin entry during the 2 h prior to mealtime (the food bin entry was also significantly increased in a 5 h window before palatable meal presentation , data not shown), relative to control mice. Anticipation of food can be manifest as either generalized locomotor activity or activity directed at a food bin, or both. In one case, food bin entry was increased whereas locomotor activity was not: a study of obese rats with hypothalamic lesions stationed themselves at the food bin prior to a daily mealtime, and exhibited anticipatory nose pokes without significant anticipatory locomotor activity [26]. Contrary to our expectation, female C57BL/6J mice did not show anticipatory activity or food bin entry when receiving a daily high fat meal. This could be because the female received the high fat at an earlier time in the light cycle than did the male mice. Interestingly, when the daily high fat meal was not delivered, the female mice reacted by subtly increasing activity and food bin entry at expected meal time for two days, suggesting that these mice did indeed anticipate food arrival (or were entrained rhythmically) but did not express anticipatory activity or food bin entry. A similar phenomenon was described for rats receiving daily drug injections; these animals did not show anticipatory activity but did show a strong increase in activity at and beyond the time of drug delivery when the drug was withdrawn [29].

The difficulty in inducing anticipation of a daily palatable meal is underscored by our results which comprise separate cohorts of C57BL/6J mice on palatable meal schedules, tested in separate laboratories utilizing different measurement devices. The fact that one condition tested (high fat diet in males) elicited subtle anticipation in terms of food bin entry and activity demonstrates the value of more refined behavioral analyses in evaluating the ability of wild-type mice (and, potentially, mice with neural or genetic defects, e.g., [25]) to anticipate a daily opportunity to eat a preferred food. Due to its cost efficiency, reproducibility, and throughput, wheel running is an excellent metric for food anticipation during restricted access to normal chow, but wheel running in mice is very strongly influenced by daily caloric intake, as evidenced by the rapid induction of continuous running when food was removed overnight in Experiment 1 (Group 1), and the immediate and complete loss of pre-meal running when the mice were transitioned from a caloric restriction schedule with daytime feeding to an ad libitum food access schedule with a daytime palatable snack.

All 8 food restricted mice in Group 1, Experiment 1, showed very low levels of wheel running on the first night after an overnight fast and the first mid-day feeding. Nocturnal running reappeared on the second night, but several hours later than usual. Running onset then advanced each night, reestablishing a normal phase within 5–7 days. This change in the timing of nocturnal activity could represent an acute phase shift of the light-entrained circadian pacemaker, induced by some correlate of food deprivation or refeeding, such as hyperactivity prior to the first meal or hypoactivity after the first meal, behaviors that are known to shift circadian rhythms in rodents. Additional studies will be necessary to test this hypothesis, and identify the phase resetting stimulus if a pacemaker shift is confirmed. Changes in nocturnal activity were also evident in the other groups, primarily in the amount of running during the second half of the night, which tended to decline over time. We did not include a group of mice maintained on regular chow without palatable snacks, but it is common to see a decrease in total nocturnal activity in rodents over many weeks of recording, so changes in waveform may be unrelated to the palatable feeding schedules.

An unexpected difference between Experiments 1 and 2 was in the total caloric intake of mice during scheduled access to a palatable food. Both experiments included at least one group of mice with restricted daytime access to milk chocolate and free access to regular chow. Under these conditions, mice with running wheels exhibited a gradual increase in body weight while mice without wheels in the video analysis study exhibited a gradual decrease in total daily caloric intake and body weight. The reason for this difference is unclear, but may be related to differences in initial starting weights (∼23 g vs ∼27 g, in Experiments 1 and 2, respectively), in the amount of chocolate eaten (∼1.4 g vs 0.9 g, in Experiments 1 and 2, respectively), and/or in the timing of the palatable meal (8 h vs 2 h prior to lights-off, in Experiments 1 and 2, respectively). The difference in the amount of chocolate eaten no doubt contributes to the body weight differences, but the timing of the meals may also be important. In Experiment 1, the palatable snack stimulated food intake in the middle of the light period, a time of day in nocturnal rodents when intake of regular chow and total energy expenditure (locomotor activity and basal metabolism) is normally low. Increased food intake in the middle of the rest phase may favor weight gain, and is associated with obesity induced by cafeteria diets, lesions, and gene mutations [27], [28]. In Experiment 2, the palatable snack was provided in the last 2 h of the light period, when spontaneous food intake and basal metabolism are normally increasing prior to lights-off and the onset of the daily active phase. A palatable snack at that time displaces chow intake, and may also create a ‘reward contrast’ effect, resulting in a reduction in the perceived palatability of regular chow, and a consequent reduction in total chow intake, promoting gradual weight loss rather than weight gain. A reward contrast effect might also account for the delayed appearance and lower magnitude of food anticipatory wheel running in Experiment 1 (Group 1) mice during their second food restriction schedule, after 4 weeks of receiving a palatable snack at that time of day.

The efficacy of a high fat rodent chow (∼36% fat and 36% carbs) relative to chocolate (26% fat, 67% carbs) as a stimulus for anticipatory food-bin entry and mild anticipatory activity was surprising, given previous work with rats demonstrating that a high carbohydrate meal is a more effective stimulus than is a high fat meal for resetting of food-anticipatory rhythms by a shift of mealtime [30]. The palatable meal studies in rats also suggest that the caloric load provided by a daily snack was critical in determining whether anticipatory activity emerges [26]. Since the caloric content of the chocolate and high fat meal was similar and the total chow intakes were nearly identical in chocolate or high fat fed mice, the critical variable for inducing anticipatory food bin approaches in mice and mild food anticipatory activity may be related to neural or endocrine responses to fat intake. This could include correlates of palatability, given that mice ate significantly more high fat food when given a choice between high fat and chocolate. Despite the absence of anticipatory activity to some snacks known to be palatable, these results establish guideposts by which palatable meal anticipation can be studied further in mice, thereby expanding the genetic tools available for interrogating the neurobiology of anticipation.
